# Review and results of a survey about biosimilars prescription and challenges in the Middle East and North Africa region

**DOI:** 10.1186/s40064-016-3779-8

**Published:** 2016-12-30

**Authors:** Fadi Farhat, Ahmad Othman, Fadi el Karak, Joseph Kattan

**Affiliations:** 1 Department of Hematology-Oncology, Hammoud Hospital University Medical Center, G. Hammoud Street, 652, Sidon, Lebanon; 2 Faculty of Medicine, Saint Joseph University, Beirut, Lebanon; 3 Department of Medical Oncology, Hôtel-Dieu de France University Hospital, Beirut, Lebanon

**Keywords:** Biosimilars, Lebanon and Arab world markets, Prescription, Pricing

## Abstract

**Background:**

Only drafts of regulatory guidelines for the registration of biosimilars are available in Lebanon. We analyzed the results of a regional survey conducted in Lebanon to understand the impact of different parameters on the acceptance and future prescription of biosimilars. We also reviewed the current situation of biosimilars around the world. The study surveyed healthcare professionals from the Arab countries, Iran, Belgium and Italy. Data about the participants’ specialty, country of residence, their knowledge about biosimilars, biosimilars’ prescription, price influence and the manufacturer’s credibility were collected.

**Results:**

117 questionnaires were completed and returned: 46 (39.3%) respondents were oncologists. 72 (61.5%) respondents were Lebanese, and the others from Egypt, Syria, Algeria, Iraq, Sudan, Jordan, Iran, Belgium and Italy. 77 (65.8%) respondents had knowledge about biosimilars, of whom 48 (62.3%) considered biosimilars as biologics that demonstrate bioequivalence with the original biodrug and have all preclinical and clinical trials equal to those already performed with the original biodrug. 74 (63.2%) out of 117 respondents agreed that biosimilars in the Arab and Middle Eastern market are already marketed. Among the 48 participants who prescribe biosimilars, the main prescription driver was the drug’s approval by the FDA and EMA (68.8%). 71 (60.7%) respondents considered that the main advantage of biosimilars is their lower price and 41 (35%) out of the 117 respondents declared that they should know in which country the drug has been tested/created before using it in their own country. 35% of the respondents thought that the cost of a treatment should not come before its effectiveness or safety/tolerance, given that the biosimilar will be less expensive than the reference drug.

**Conclusions:**

Biosimilars’ acceptance and use is increasing worldwide. Only few physicians are aware of biosimilars presence in the market and do prescribe them in Lebanon and the Arab region. This could be mainly explained by lack of confidence in efficacy, safety, manufacturing process and price of these products, and lack of clear legislation. Thus, WHO is finalizing a new guideline for similar biotherapeutic agents. This could be a starting point for the Lebanese government to support the authorization of biosimilars.

**Electronic supplementary material:**

The online version of this article (doi:10.1186/s40064-016-3779-8) contains supplementary material, which is available to authorized users.

## Background

Introduced into the market more than 30 years ago, biological products result from biologic material and encompass a wide range of elements such as hormones, vaccines, antibodies, proteins, polysaccharides, polynucleotides, growth factors, blood products, and live viral material; they differ greatly from chemical medicines (Liang and Mackey 2012). Biosimilars are medicinal products with a similar but not identical safety, efficacy and quality as the authorized reference (originator) biologic agents. They are composed of an analogous active substance but are large molecular-weight, complex molecules that are produced in living cells through genetic engineering (Sylvester et al. 2013). They are also referred to as biological and biotherapeutic agents/products.

The Biologics Price Competition and Innovation Act (BPCIA) defines biosimilar as *a biological product highly similar to the reference product notwithstanding minor differences in clinically inactive components*; and with *no clinically meaningful differences between the biologic product and the reference product in terms of the safety, purity, and potency of the product* (FDA 2009).

In this paper, we report the results of a regional survey to understand the impact of different parameters on the acceptance and future prescription of biosimilars in the Lebanese and Arab markets. We also analyze the current situation in the region and discuss the worldwide situation of biosimilars focusing mainly on the European Union (EU) and the United States (U.S.) laws, regulations and legislation pathways, pricing and challenging market access.

## Methods

A regional survey was conducted from 26 to 28 March 2015 during the Pan Arab Oncology Meeting in Beirut, Lebanon. The meeting drew over 150 participants from the Arab countries, Iran, Belgium and Italy. It was attended by a wide range of healthcare professionals: oncologists, hematologists, hemato-oncologists, gynecologist, surgeons, pulmonologists, radiologists, anatomopathologists, pharmacists and others.

A two-page questionnaire of 14 questions was distributed to all the meeting participants. The survey tool collected data about the participants’ specialty, country of residence, their knowledge about biosimilars, biosimilars’ prescription, price influence and the manufacturer’s credibility (Additional file 1). Data collected were described with frequencies and percentages using the SPSS software application (IBM Corp. Released 2014. IBM SPSS Statistics for Windows, Version 23.0. Armonk, NY: IBM Corp).

## Results

In total, 117 questionnaires were completed and returned. The results of the survey showed that 46 (39.3%) respondents were oncologists, 17 (14.5%) were hemato-oncologists and 14 (12%) were nurses while only 2 (1.7%) were hematologists. The majority of the respondents were Lebanese (61.5%, n = 72), 12% were from Egypt, 11.1% from Syria and the others from Algeria, Iraq, Sudan, Jordan, Iran, Belgium and Italy (Table 1).

**Table 1:** Specialty and country of residence of the survey participants (n = 117)

Variable	N (%)
Specialty	
Oncology	46 (39.3)
Hemato-oncology	17 (14.5)
Nurse	14 (12.0)
Surgery	10 (8.5)
Gynecology	6 (5.1)
Pharmacy	6 (5.1)
Urology	5 (4.3)
Radiology	3 (2.6)
Hematology	2 (1.7)
Anatomopathology	1 (0.9)
Other	7 (6.0)
Country of residence	
Lebanon	72 (61.5)
Egypt	14 (12.0)
Syria	13 (11.1)
Algeria	8 (6.8)
Iraq	3 (2.6)
Belgium	2 (1.7)
Sudan	2 (1.7)
Jordan	1 (0.9)
Iran	1 (0.9)
Italy	1 (0.9)

Moreover, 77 (65.8%) out of the 117 respondents had knowledge about biosimilars. Among the 77 respondents who seemed to have a good understanding of scientific issues pertaining biosimilars, 48 (62.3%) respondents considered biosimilars as biologics that demonstrate bioequivalence with the original biodrug and have all preclinical and clinical trials equal to those already performed with the original biodrug. Besides, when approved, they already have a well-defined immunogenicity.

As for biosimilars’ prescription, 74 (63.2%) out of 117 respondents agreed that biosimilars in the Arab and Middle Eastern market are already marketed. In parallel, 53 (45.3%) respondents agreed that biosimilars are being manufactured in the same region. In addition, 48 (41%) respondents confirmed that they prescribe biosimilars versus 38 (32.5%) who do not prescribe them. Among the 48 participants who prescribe biosimilars, the main driver for prescribing them was the drug’s approval by the Food and Drug Administration (FDA) and the European Medicines Agency (EMA) (68.8%), followed by a lower price of the bioequivalence in comparison with the innovator (64.6%), then bioefficacy (47.9%), safety (41.7%), good manufacturing practices and high reputation of the manufacturer (31.3%).

As for the price influence, 35% of the respondents thought that the cost of a treatment should not come before its effectiveness or safety/tolerance, given that the biosimilar will be less expensive than the reference drug. In contrast, 25.6% thought that the lower prices of the biosimilar are a good news because patients will be treated with biologics, and would help cost savings (26.5%).

The results also showed that physicians would mainly trust a company highly experienced in manufacturing small-molecule generic drugs as a producer of biosimilars (48.7%), knowing that they have the expertise to deal with regulatory authorities and have the knowledge of approval’s guidelines. Interestingly, physicians will trust more a company with prior experience in manufacturing biologics as a manufacturer of biosimilars (54.7%) (Table 2).

**Table 2:** Knowledge about biosimilars and their prescription (n = 117)

Questionnaire item	N (%)	N (%)
Knowledge about biosimilars		
Do you know what biosimilars are?		
Yes	77 (65.8)	
No	14 (12.0)	
No answer	26 (22.2)	
Choose one item that adjusts to your concept of biosmilars^a^		
A biologic that demonstrates bioequivalence with the original biodrug and has all preclinical and clinical trials equal to those already performed with the original biodrug. Besides, when approved, it already has a well-defined immunogenicity	48/77 (62.3)	
A biologic that demonstrates bioequivalence with an original biodrug and does not need clinical trials to be commercialized	12/77 (15.6)	
A molecule equal to that of the original biologic but of lower production cost	17/77 (22.1)	
An attempt to copy an innovative biodrug and will never be equal to it	1/77 (1.3)	
A generic biologic of an already commercialized biodrug	4/77 (5.2)	
Biosimilars’ prescription		
Do you agree with the information that there are already marketed biosimilars in the Arab and Middle Eastern Market?		
Yes	74 (63.2)	
No	22 (18.8)	
No answer	21 (17.9)	
Do you agree with the information that biosimilars are being manufactured in the Arab and Middle Eastern Market?		
Yes	53 (45.3)	
No	38 (32.5)	
No answer	26 (22.2)	
Do you prescribe biosimilars?		
Yes	48 (41.0)	
No	38 (32.5)	
No answer	31 (26.5)	
What are the major drivers that encourage you to prescribe biosimilars?		
Safety	20/48 (41.7)^b^	40/117 (34.2)^c^
Bioefficacy	23/48 (47.9)^b^	45/117 (38.5)^c^
FDA and EMA approval for biosimilar	33/48 (68.8)^b^	61/117 (52.1)^c^
Good manufacturing practices and high reputation of the manufacturer	15/48 (31.3)^b^	31/117 (26.5)^c^
Country of origin of the biosimilars’ manufacturer	14/48 (29.2)^b^	25/117 (21.4)^c^
Lower price of the bioequivalence in comparison with the innovator	31/48 (64.6)^b^	58/117 (49.6)^c^
Nothing encourages you	2/48 (4.2)^b^	6/117 (5.1)^c^
What are the major local drivers that encourage you to prescribe biosimilars?		
Assurance that phase III clinical trials will be performed in a sample of the local population	14/48 (29.2)^b^	33/117 (28.2)^c^
Maintenance of an adequate national system of pharmacovigilance specific to biosimilars	23/48 (47.9)^b^	36/117 (30.8)^c^
Transparency of the local health regulatory authority(ies)	16/48 (33.3)^b^	34/117 (29.1)^c^
Nothing encourages you	7/48 (14.6)^b^	14/117 (12.0)^c^
In your opinion, what are the advantages of a biosimilar?		
Lower price	71/117 (60.7)	
Commercialization approved with initial indication including all diseases previously approved for the innovative biodrug	32/117 (27.4)	
Administration route different from that of the original biodrug	10/117 (8.5)	
Lower therapeutic dose	8/117 (6.8)	
There are no advantages	6/117 (5.1)	
Now that biosimilars are coming to the market, you think that^d^		
Patient associations should be informed and should be able to give their opinion	28/117 (23.9)	
Patients should systematically be given information	28/117 (23.9)	
We should wait for many patients to receive biosimilars in a real life setting before recommending its use in a large population of patients	16/117 (13.7)	
We should know in which country the drug has been tested/created before using it in your own country	41/117 (35.0)	
Price influence		
The biosimilar will be less expensive than the reference drug, you think that^d^		
These are good news because more patients will be treated with biologics	30/117 (25.6)	
The cost of a treatment should not come before its effectiveness or safety/tolerance	41/117 (35.0)	
This will help cost savings	31/117 (26.5)	
If biosimilars are already in the market, a 30% reduction from the innovator’s price will be sufficient	15/117 (12.8)	
You do not think that a lower cost will change something	4/117 (3.4)	
Manufacturers’ credibility		
Do you trust a company highly experienced in manufacturing small-molecule generic drugs as a producer of biosimilars knowing that they have the expertise to deal with regulatory authorities and have the knowledge of approval’s guidelines?		
Yes	57 (48.7)	
No	19 (16.2)	
No answer	41 (35.0)	
Do you trust a company with prior experience in manufacturing biologics as a manufacturer of biosimilars?		
Yes	64 (54.7)	
No	13 (11.1)	
No answer	40 (34.2)	

^a^Some people who said no to knowledge also answered this question which is not correct since it says in the questionnaire that only those who said yes in the prior question should answer these questions. Those people who answered but they should have not were not included in this table. For those eligible to answer, they should choose one answer but some of them did more than one question (hence the overall % will exceed 100%)

^b^Answers among respondents who answered yes to prescribing biosimilars

^c^Answers among all respondents

^d^Participants were supposed to pick only one answer but they did not

## Discussion

Our survey marks the acceptance of physicians to a biosimilar submitted and responding to EMA and FDA regulations. Our finding complies with international results where PubMed-indexed manuscripts and grey literature reported the acceptance of prescribing FDA-approved biosimilars by almost all physicians surveyed. These physicians also believe that the FDA-approved biosimilars have the same efficacy (effectiveness) and safety profile as the originator biological (Felix et al. 2014).

Pricing is still a key factor and cost saving also seemed to be an important driver for prescribing biosimilars (26.5%). We note that some biosimilars are marketed at a reduced price, compared to the innovator. Such low price can only be explained by a trivial investigational cost, thus the absence of adequate comparative studies. Consequently, efficacy and safety of such drugs are questionable. EMA and FDA commonly reject these drugs for lack of scientific criteria of safety. Recognition and authorization from legal entities is crucial for physicians, to encourage them to prescribe biosimilars, but what will influence the prescriber? Is it patients, fellows or the health system? In fact, the major factor influencing the prescriber is the payer. This is the case in organizations where the health authorities can force the prescriber, and where the substitution of the innovator is applied. Thus, this proves the doubt in biosimilars efficacy and safety by physicians in addition to the unwillingness to prescribe biosimilars unless mandatory. Ultimately, the credibility of the biosimilar is the main driver for the launch of any biosimilar in the Arab world. While literature reports that the manufacturers may use higher rebates on originator biologicals as a barrier to adopting biosimilars (Felix et al. 2014), we aim to investigate in future surveys what prescribing behavior would have changed had the price levels been specifically examined. Also, our next survey will focus on understanding how Lebanese and Arab health care professionals perceive an adequate level of “trust” in the medication, namely how much a discount makes this affordable and testing whether prescribers are aware of the pricing/payor factors in their own regions as regards to biosimilars.

In the following sections, we summarize the main points about the biosimilars’ regulations and challenging market access in USA and the European countries as well as Lebanon.

### Biosimilars worldwide

#### Regulations and laws

The U.S., EU, Japan, Canada and the pharmerging markets (China, India, Brazil and Mexico) form geographically the market for biologics and biosimilars. Each of these markets has its own regulations and laws concerning biosimilars. EU has led the way in developing a legal framework for the approval of biosimilar products via EMA.

As for the U.S., several conditions make the US as the biggest opportunity for biosimilars to extremely progress by 2020 such as the presence of competing and leading manufacturers (Pfizer and Merck), patients and health insurers needing the access to low-cost, high-value drugs. Thus, this opportunity will be the cut-off point for the success or failure of biosimilars in the next decade (Kresse 2009; IMS Health 2011).

##### EU guidelines

EMA is held accountable for the scientific assessment of medicines for use in EU countries, and for the approval of all medicines for human and animal use derived from biotechnology and other high-tech processes including biosimilars. Those guidelines concerning biosimilars’ requirements for the market approval have been dictated by the EMA in accordance with the funds of the Community Code. They enclose the following: (1) the original product must be authorized within the EU, (2) the 8 years data exclusivity of the reference must be expired, (3) the similarity between the safety, efficacy and quality of the biosimilar and the original biologic product must have been demonstrated, (4) the primary amino acid arrangement of the biosimilar protein has to be matching to the reference product amino acid sequence, (5) the biosimilar manufacturer needs to validate each step of development, comparative but possibly abbreviated non-clinical pharmacology and toxicology studies, human Pharmacokinetics (PK) and Pharmacodynamics (PD) studies and human efficacy and safety studies including immunogenicity, and (6) the Risk Management Plan must include pharmacovigilance (IMS Health 2011; Church et al. 2009; Choy and Jacobs 2014).

Since October 2006 till present, EMA has approved 21 biosimilars corresponding to 8 active molecules, in six categories of biologics (epoetins, filgrastims, follitropins, growth hormones, insulins, and monoclonal antibodies) (European Medicines Agency 2016) (Table 3).

**Table 3:** EMA approved biosimilars (European Medicines Agency 2016)

Date of biosimilar EMA approval	Biosimilar product	Original product	Active substance
12 April 2006	Omnitrope	Genotropin	Somatropin
28 August 2007	Abseamed	Epogen/Eprex	Epoetin alfa
28 August 2007	Binocrit	Epogen/Eprex	Epoetin alfa
28 August 2007	Epoetin Alfa Hexal	Epogen/Eprex	Epoetin alfa
18 December 2007	Silapo	Epogen/Eprex	Epoetin zeta
18 December 2007	Retacrit	Epogen/Eprex	Epoetin zeta
15 September 2008	Biograstim	Neupogen	Filgrastim
15 September 2008	Ratiograstim	Neupogen	Filgrastim
15 September 2008	Tevagrastim	Neupogen	Filgrastim
06 February 2009	Filgrastim Hexal	Neupogen	Filgrastim
06 February 2009	Zarzio	Neupogen	Filgrastim
08 June 2010	Nivestim	Neupogen	Filgrastim
10 September 2013	Inflectra	Remicade	Infliximab
27 September 2013	Ovaleap	Gonal-f	Follitropin alfa
10 October 2013	Remsima	Remicade	Infliximab
18 October 2013	Grastofil	Neupogen	Filgrastim
27 March 2014	Bemfola	Gonal-f	Follitropin alfa
09 September 2014	Abasaglar (previously Abasria)	Lantus	Insulin glargine
18 September 2014	Accofil	Neupogen	Filgrastim
14 January 2016	Benepali	Enbrel	Etanercept
26 May 2016	Flixabi	Remicade	Infliximab

Moreover, substitutability and interchangeability differ from a country to another in the EU, each having implemented its own legislation. For instance, France and Italy exclude substitution of innovative biologics by biosimilars, United Kingdom (UK) restricts these substitutions. Hence, the European biopharmaceutical industries are asking for demonstration and a clear differentiation of the concepts of similarity and interchangeability (IMS Health 2011).

##### U.S. guidelines

Since 2006, the FDA has approved biological products based on comparability such as Omnitrope, Enoxatrapine and Genotropin. It is only in 2010, after obtaining the Patient Protection and Affordable Care (PPAC) Act signed by President Obama, that the US had the authority to approve biosimilars (FDA 2010). The 2009 BPCIA formally passed under the PPAC Act, an amendment of the Public Health Service Act (PHS Act) reduced approval pathway for biological agents that showed to be greatly similar (biosimilar) to a FDA approved biological product. The BPCIA is theoretically comparable to the Drug Price Competition and Patent Term Restoration Act of 1984 that created biological drug approval through the Federal Food, Drug, and Cosmetic Act (FFD&C Act). The BPCIA supports the FDA’s long-lasting policy that allows adequate trust on the known information about a drug, thus saving time, resources and avoiding the repetition of already available human or animal tests (Nick 2012).

Another important part of the amendment in the PPAC Act for biosimilars is the exclusivity of the data. Data exclusivity is the time frame between biosimilar filling on the innovator data. It is designed to protect the innovation taking into consideration the time-consuming, expensive and uncertain process involved during the waiting period of the innovator to gain the FDA approval. The data exclusivity time ranges between 12 and 14 years (US Code 2012).

In April 2015, the FDA released 2 highly-anticipated guidances on biosimilars. The first guidance “Scientific Considerations in Demonstrating Biosimilarity to a Reference Product” assists companies in demonstrating that a proposed therapeutic protein product is biosimilar to a reference product for the purpose of submitting an application, called a “351(k)” application, to the FDA. It also describes a risk-based “totality-of-the-evidence” approach that the FDA intends to use to evaluate the data and information submitted in support of a determination of biosimilarity of the proposed product to the reference product (U.S. Department of Health and Human Services et al. 2015a). The second guidance entitled “Quality Considerations in Demonstrating Biosimilarity to a Reference Protein Product” provides an overview of analytical factors to consider when assessing biosimilarity between a proposed therapeutic protein product and a reference product for the purpose of submitting a 351(k) application. This includes the importance of extensive analytical, physico-chemical and biological characterization in demonstrating that the proposed biosimilar product is highly similar to the reference product notwithstanding minor differences in clinically inactive components (U.S. Department of Health and Human Services et al. 2015b). Also in May 2015, the FDA released a draft guidance on “Biosimilars: Questions and Answers Regarding Implementation of the Biologics Price Competition and Innovation Act of 2009”. This guidance document is being distributed for comment purposes only and provides answers to common questions from people interested in developing biosimilar products. The question and answer format addresses questions that may arise in the early stages of product development, such as how to request meetings with the FDA, addressing differences in formulation from the reference product, how to request exclusivity, and other topics (U.S. Department of Health and Human Services et al. 2016).

As of 05 October 2016, the FDA authorized 4 biosimilars (Table 4).

**Table 4:** FDA approved biosimilars

Date of biosimilar FDA approval	Biosimilar product	Original product	Active substance
06 March 2015 (U.S. Department of Health and Human Services and Food and Drug Administration 2016a)	Zarxio	Neupogen	Filgrastim
05 April 2016 (U.S. Department of Health and Human Services and Food and Drug Administration 2016b)	Inflectra	Remicade	Infliximab
30 August 2016 (U.S. Department of Health and Human Services and Food and Drug Administration 2016c)	Erelzi	Enbrel	Etanercept
23 September 2016 (U.S. Department of Health and Human Services and Food and Drug Administration 2016d)	Amjevita	Humira	Adalimumab

#### Pricing and challenging market access

Biosimilars’ sales are projected to reach around $25–$35 billion by 2020. Since the first biosimilar approval in EU in 2006, 21 biosimilars are now authorized by the EMA (European Medicines Agency 2016) (Table 3), 4 biosimilars by the FDA (Table 4), and 250 biosimilars are in the pipeline globally (Deloitte 2015). This market is growing tremendously in spite of the effort to reduce the global healthcare costs, which is making it the fastest rising market of all biologics sector in the next 5 years (IMS Health 2011).

This increase in spending was estimated to 700% between 2010 and 2015, summing up to approximately $80 billion in global sales to replace branded biologic agents losing their patent by 2015 (Liang and Mackey 2012). Consequently, switching to biosimilars would help in saving billions of dollars annually. This is expected to reach $40–$250 billion in the coming 10 years in the U.S. alone (Deloitte 2015). However, the expected success and progress of biosimilars in Europe is higher than the US markets due to the presence of general and specific guidelines. Other factors such as comparability studies (quality, efficacy and safety), substitution, pricing and reimbursement may affect biosimilars market accessibility (Simoens et al. 2011). Thus, we assume that switching to biosimilars does not consider “biobetter” prescribing, where a better biologic will still be priced higher, and prescribers will likely lean toward the more effective biologic.

Moreover, due to the high research and developmental cost of biosimilars, the difference of prices between them and the reference biological agent is supposed to be less compared to the reference and generics. In fact biosimilars are priced 30% less than the originator compared to a reduction of 80% for the generics after the first 6 months to years after its entry to the market. Although this reduction may not be significant but seems to be sufficient to generate high profitable rates (Lockwood et al. 2013).

However, the biosimilar market is not easy to access given the challenges in terms of regulatory uncertainty production complexity, interchangeability and competition. In particular, the lack of clear guidelines on interchangeability and substitutability with reference biologics will probably cause physicians to be more cautious in prescribing biosimilars until they gain comfort with the efficacy and quality of biosimilars (Deloitte 2015). These entry barriers for manufacturers should be crossed to penetrate a highly competitive platform, yet cost-effective. Thus, for a positive entry to the biosimilar market, few major abilities should be accomplished such as adequate research and development capabilities, specific biomanufacturing platforms, legal expertise and international distribution channels, global network of marketing sales and a robust lobbying with regulatory organizations, opinion leaders, governments and key people to facilitate the approval of laws and regulations (Calo-Fernandez and Martinez-Hurtado 2012).

### Biosimilars in Lebanon

#### Indications and consumption

Lebanon’s healthcare sector is considered as one of the best in the Middle East and North Africa (MENA) region. Many biosimilars are being used, as in the global market, such as granulocyte colony stimulating factor (G-CSF) frequently prescribed in the hemato-oncology field. The list also includes erythropoietin given to patients with chronic renal failure or oncological ailments, Humira^®^, Remicade^®^ and Enbrel^®^ in rheumatoid arthritis, psoriasis, and other immunological diseases.

A 2006 report by the World Health Organization (WHO) about the Health System Profile in Lebanon showed that the total health expenditure of Gross Domestic Products is about 12% (World Health Organization 2006). However, being expensive therapeutic drugs, biosimilars are not accessible for all Lebanese population. Hence, major imported oncological drugs to the Lebanese market were studied in terms of cost and consumption to show their impact on total budget.

#### Guidelines: laws and regulations

To assure the biosafety and efficacy of biosimilars, the Lebanese Ministry of Public Health (MOPH) decided to develop requirements and guidance for registration of biosimilar drugs. Those were drafted in 2013 and follow the EMA guidelines, WHO and International Conference for Harmonization (ICH) Guidance on Similar Biological Products in collaboration with the French National Agency for Medicines and Health Products safety (*Agence Nationale de Securité des Médicaments*, ANSM) (CTD format) (Republic of Lebanon 2013). Consequently, a special committee to propose amendments to the decree 571/2008 concerning the regulatory guidelines for biosimilar medicinal products in Lebanon was formed. While waiting for the committee approval and an update of legal texts, the approval of biosimilars follows same rules as generics. Neither the “Requirements for Drug Substance and Finished Product Checklist” nor the “Requirements for Bioequivalence Study Checklist” requested analysis specific to the biosimilars are present. Biosimilars, as separate category is not even mentioned for the time being.

The “General Regulatory and Manufacturing Rules” state in the decree 571 that “the international authorities that should be followed for approval of registration are the FDA, the American Medical Association (AMA) and WHO. The drug that will be approved should also be registered in at least 2 countries”. Based on the following decision of the MOPH, prices are established based on the price in the country of origin with comparison to the established median price in many European countries such as France, UK, Belgium, Switzerland, Italy, Spain and Portugal. But nothing is mentioned concerning the pricing of biosimilars per se.

#### Challenges

Every country in the Arab world has a different health system and has its own laws concerning registration of new drugs. Drug patent, protecting brands and innovators differ as well. For instance in Jordan, any generic can be introduced in the Jordanian market after 3 years of launching in the country where it was first approved (Jordan Food and Drug Administration 2016), while in Lebanon the drug’s patent follows the international guidelines. This difference can be explained by the fact that not all countries are member of the World Trade Organization (WTO).

With the approaching patent expiration of major biopharmaceutical products and the emerging growth of biosimilars in global markets, issues will arise due to the fact that biological products or biosimilars are different from the small generic drugs. Safety concerns will surface again unless biosimilars prove to have a comparable safety profile. Alike other countries, prescriptions of biosimilars in Lebanon and the Arab world will face many challenges, especially the patient and physician’s trust in the medication per se, the affordability, accessibility and availability issues.

## Conclusions

Biosimilars’ acceptance and use is growing worldwide. Despite this accomplishment, only the EU via EMA and US FDA have dictated specific regulations and guidelines related to biosimilars. Only few physicians are aware of biosimilars presence in the market and do prescribe them. Further investigations must be carried out to find out whether this is ascribed to lack of confidence in efficacy, safety, manufacturing process and price of these products. Lack of clear legislation is likely to be responsible for the low rate of awareness about biosimilars; thus, WHO is finalizing a new group of guidelines for similar biotherapeutic agents. This is intended to help national regulatory agencies, manufacturers, and other interested parties and could be a starting point for the Lebanese government to support the authorization of biosimilars.

## Additional file

**Additional file 1.** Survey questionnaire.

## Abbreviations

AMA: American Medical Association
ANSM: *Agence Nationale de Securité des Médicaments* (French National Agency for Medicines and Health Products safety)
EMA: European Medicines Agency
EU: European Union
FDA: Food and Drug Application
FFD&C: Federal Food, Drug, and Cosmetic
G-CSF: granulocyte colony stimulating factor
ICH: International Conference for Harmonization
MENA: Middle East and North Africa
MOPH: Ministry of Public Health
PD: pharmacodynamics
PHS: Public Health Service
PK: pharmacokinetics
PPAC: Patient Protection and Affordable Care
UK: United Kingdom
U.S.: United States
WHO: World Health Organization
WTO: World Trade Organization
