# Supplementary material for: Review and results of a survey about biosimilars prescription and challenges in the Middle East and North Africa region
Source: Springerplus. 2016 Dec 30;5(1):2113. doi: 10.1186/s40064-016-3779-8 (PMC5201599; doi:10.1186/s40064-016-3779-8)
Supplement: Supplementary file 1 — Additional file 1. Survey questionnaire. [file 40064_2016_3779_MOESM1_ESM.docx]

| 1. **Specialty** |
| --- |
| Oncologist  Hematologist  Onco-hematologist  Gynecologist  Surgeon  Pulmonologist  Radiologist  Anatomopathologist  Pharmacist  Other, please specify: ………………………… |
| 1. **Country of residence** |
| Lebanon  Jordan  Palestine  Syria  Iraq  Bahrain  KSA  Kuwait  Oman  Qatar  UAE  Yemen  Algeria  Egypt  Lybia  Morocco  Tunisia  Iran  Turkey  Other, please specify: ………………………… |
| 1. **Knowledge about biosimilars** |
| **3.1.** **Do you know what biosimilars are?**  Yes  No |
| **3.2. In case of an affirmative answer, choose one item that adjusts to your concept of biosimilars:**   \| A biologic that demonstrates bioequivalence with the original biodrug and has all preclinical and clinical trials equal to those already performed with the original biodrug. Besides, when approved, it already has a well-defined immunogenicity. \|  \| \| --- \| --- \| \| A biologic that demonstrates bioequivalence with an original biodrug and does not need clinical trials to be commercialized \|  \| \| A molecule equal to that of the original biologic but of lower production cost \|  \| \| An attempt to copy an innovative biodrug and will never be equal to it \|  \| \| A generic biologic of an already commercialized biodrug \|  \| |
| 1. **Biosimilars prescription** |
| **4.1. Do you agree with the information that there are already marketed biosimilars in the Arab and Middle Eastern Market?**  Yes  No |
| **4.2. Do you agree with the information that biosimilars are being manufactured in the Arab and Middle Eastern Market?**  Yes  No |
| **4.3.** **Do you prescribe biosimilars?**  Yes  No |
| **4.4. What are the major drivers that encourage you to prescribe biosimilars? More than one item can be pointed.**   \| Safety \|  \| \| --- \| --- \| \| Bioefficacy \|  \| \| FDA and EMA approval for biosimilars \|  \| \| Good manufacturing practices and high reputation of the manufacturer \|  \| \| Country of origin of the biosimilars’ manufacturer \|  \| \| Lower price of the bioequivalence in comparison with the innovator \|  \| \| Nothing encourages you \|  \| |
| **4.5. What are the major local drivers that encourage you to prescribe biosimilars? More than one item can be pointed.**   \| Assurance that phase III clinical trials will be performed in a sample of the local population \|  \| \| --- \| --- \| \| Maintenance of an adequate national system of pharmacovigilance specific to biosimilars \|  \| \| Transparency of the local Health regulatory authority(ies) \|  \| \| Nothing encourages you \|  \| |
| **4.6. In your opinion, what are the advantages of a biosimilar? More than one item can be pointed.**   \| Lower price \|  \| \| --- \| --- \| \| Commercialization approved with initial indication including all diseases previously approved for the innovative biodrug \|  \| \| Administration route different from that of the original biodrug \|  \| \| Lower therapeutic dose \|  \| \| There are no advantages \|  \| |
| **4.7. Now that biosimilars are coming to the market, you think that - Choose one of the following answers:**   \| Patient associations should be informed and should be able to give their opinion \|  \| \| --- \| --- \| \| Patients should systematically be given information \|  \| \| We should wait for many patients to receive biosimilars in a real life setting before recommending its use in a large population of patients \|  \| \| We should know in which country the drug has been tested/created before using it in your own country \|  \| |
| **5. Price influence** |
| **5.1. The biosimilar will be less expensive than the reference drug, you think that - Choose one of the following answers:**   \| These are good news because more patients will be treated with biologics \|  \| \| --- \| --- \| \| The cost of a treatment should not come before its effectiveness or safety/tolerance \|  \| \| This will help cost savings \|  \| \| If biosimilars are already in the market, a 30% reduction from the innovator’s price will be sufficient. \|  \| \| You don’t think that a lower cost will change something \|  \| |
| **6. Manufacturer’s credibility** |
| **6.1. Do you trust a company highly experienced in manufacturing small-molecule generic drugs as a producer of biosimilars knowing that they have the expertise to deal with regulatory authorities and have the knowledge of approval’s guidelines?**   Yes  No  **6.2. Do you trust a company with prior experience in manufacturing biologics as a manufacturer of biosimilars?**   Yes  No |

Thank you for your time!
